# Etymologia: botulism

**DOI:** 10.3201/eid1111.ET1110

**Published:** 2005-10

**Keywords:** etymologia, etymology, botulism

## [boch′ə-liz-əm]

Food poisoning with neurotoxicity caused by eating food contaminated with *Clostridium botulinum*. From the Latin *botulus*, “sausage,” the disease was first recognized in Germany in persons who had eaten tainted sausage and was originally called “sausage poisoning.”

Sources: Dorland’s illustrated medical dictionary. 30th ed. Philadelphia: Saunders; 2003 and Botulism in Alaska [monograph on the Internet]. [cited 2005 Aug 26]. Available from http://www.epi.hss.state.ak.us/pubs/botulism/bot_03.htm

